# Removal of a below knee plaster cast worn for 28 months: a case report

**DOI:** 10.1186/1752-1947-5-74

**Published:** 2011-02-22

**Authors:** Helen Ingoe, Sarah Eastwood, David W Elson, Claire F Young

**Affiliations:** 1 Department of Orthopaedics, Cumberland Infirmary, Newtown Road, Carlisle, Cumbria CA2 7HY, UK

## Abstract

**Introduction:**

An unusual situation in which a below knee cast was removed after 28 months is reported. To the best of our knowledge no similar cases have been reported in the literature.

**Case presentation:**

The cast was removed from the leg of a 45-year-old Caucasian woman. Significant muscle atrophy and dense skin scales were present but the underlying skin surface was relatively healthy with only small pitted 1-2 mm ulcers. No pathogenic organisms were cultured from this environment.

**Conclusion:**

It seems likely that skin can tolerate cast immobilization for prolonged duration.

## Introduction

Extremity casts are frequently applied for routine immobilization for many acute fractures. The period of immobilization varies according to the patient and the fracture. For example, a non-operatively treated tibial fracture is rarely immobilized for longer than six months. Total contact casting has been used in the treatment of Charcot's neuropathy for periods of up to one year [1]. We report a case of a below knee cast removal after 28 months.

## Case presentation

When she was 40 years old, a Caucasian woman underwent bunion surgery for pain whilst ambulating. The wounds healed without complication but she went on to develop mechanical allodynia, intermittent swelling and a bluish discoloration of the foot, consistent with a diagnosis of type 1 complex regional pain syndrome. She received many different treatments for continued pain over the subsequent years. Drug therapies using pregabalin, strong opiates and epidural analgesia were not fully successful and she was offered a below knee cast as a temporizing measure. There was no pre-existing psychiatric diagnosis but the patient developed a psychological dependence upon this cast. She was reluctant to have it removed, believing that her pain remained inadequately treated. She failed to attend several appointments at the pain clinic. When she did return, the anesthetists asked for orthopaedic assistance to remove her cast. By this point she was 45 years old and had spent the previous 28 months in the same below knee cast. She was no longer taking regular analgesia but was unable to tolerate anyone touching her leg and therefore received a general anesthetic to facilitate the cast removal.

**Figure 1:**
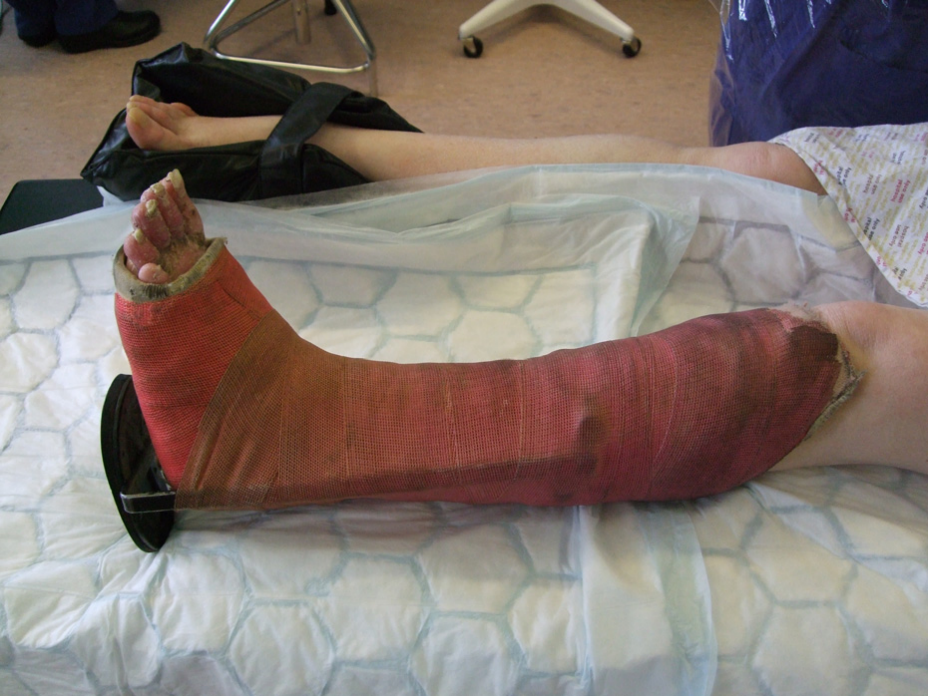
**Photograph of below knee cast prior to removal**.

**Figure 2:**
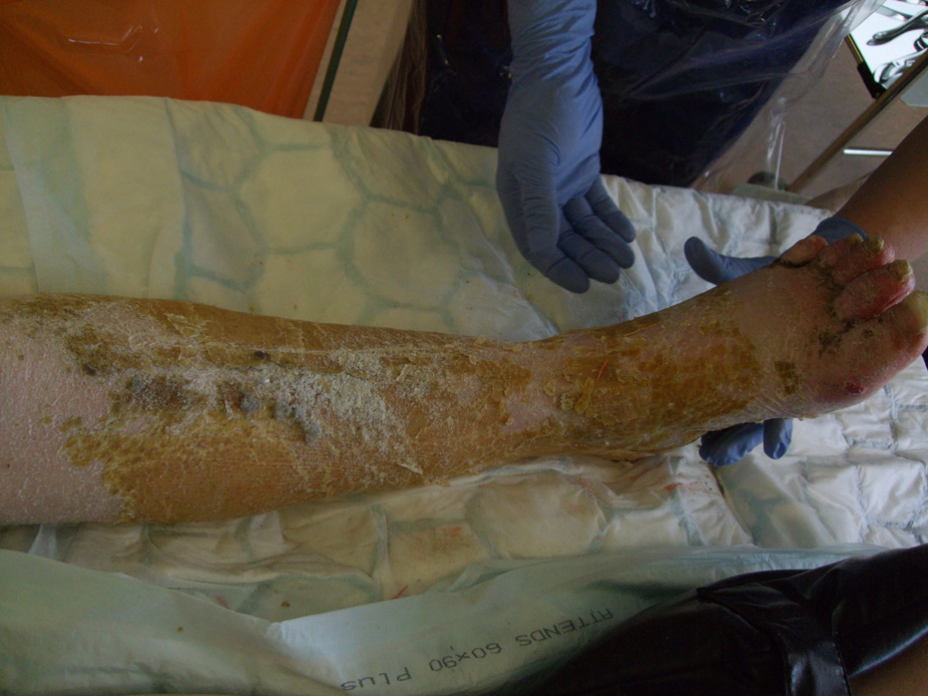
**Photograph demonstrating the appearance of leg after cast removal**.

**Figure 3:**
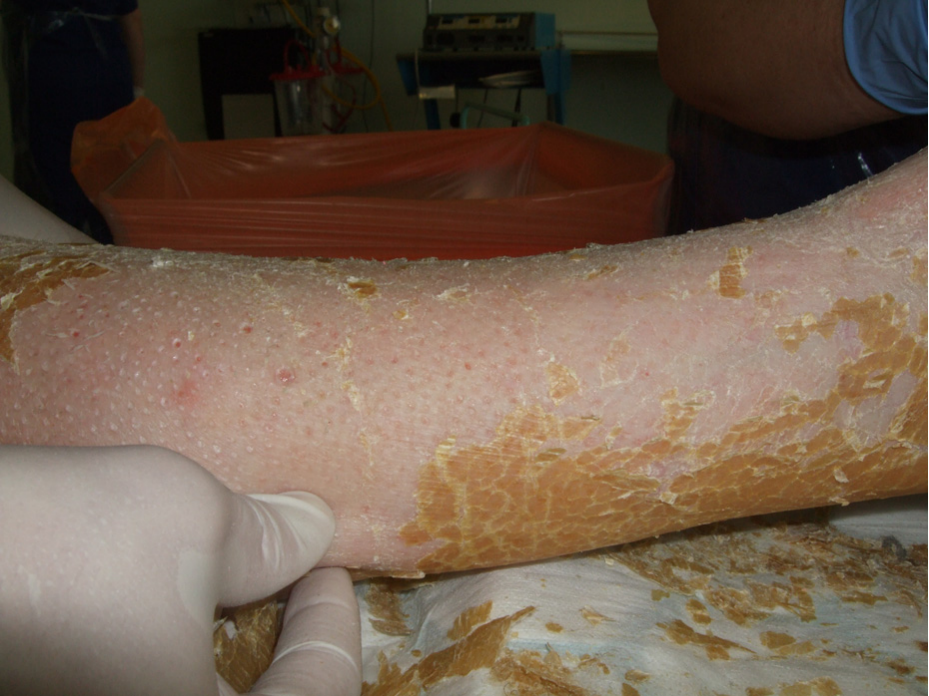
**Photograph showing yellow scales being exfoliated by hand**.

**Figure 4:**
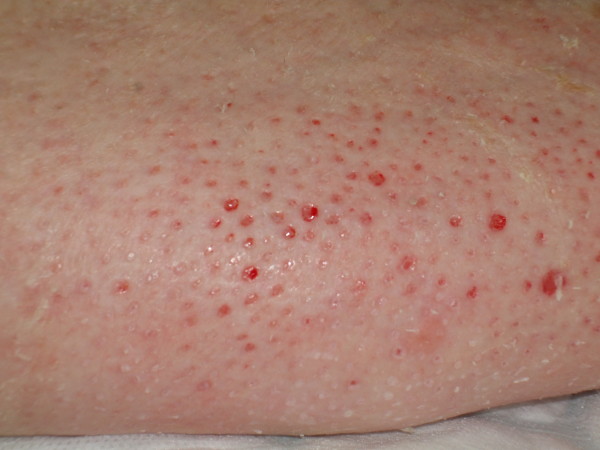
**Photograph of showing small skin pits with pink granulation tissue following removal of scales**.

The cast was found to be intact, despite having been worn for such a long period. This can be explained by the fact that she had been using crutches and the plaster was reinforced with a heel stirrup. The resin surface was filthy (Figure 1). The toes were swollen and erythematous with thick scales in the web spaces; the toenails showed evidence of onychocryptosis and onychogryphosis and had not been cut. The deep cotton bandages were intact but appeared soiled on removal of the cast. The exposed leg was covered in thick yellow skin scales (Figure 2) which were easily exfoliated by hand (Figure 3). There were no significant areas of skin loss with integument intact over bony protuberances. Dense heel callosities were removed with a sharp blade. Closer inspection of the skin surface revealed small pitted ulcers 1-2 mm in diameter replacing the normal skin pores. Healthy pink granulation tissue was seen at the base of these ulcers which appeared clean and were not infected (Figure 4). They did not bleed on palpation and required no dressing. Some superficial telangiectasia were also noted on the anterior aspect of the ankle joint which were not present elsewhere on her limbs. There was no change in skin pigmentation. The leg circumferences were reduced by 5.5 cm at the calf and 1.5 cm at the ankle when compared to the normal leg. Passive dorsiflexion was symmetrically zero degrees. Passive plantar flexion was 30° in the cast leg and 40° in the normal leg. Her passive knee movements were normal. Doppler ultrasound showed good flow at the dorsalis pedis and posterior tibial pulses. Swabs, skin and toenails sent at time of the removal of the cast showed no growth of any organisms or fungal species.

She was later reviewed in the pain clinic. Her skin was healthy but her allodynia remained symptomatic. At this stage she was reluctant to pursue any further treatment.

## Discussion

Cast immobilization is a routine orthopedic treatment which is administered for short periods of time in order to limit its complications. Total contact casts are used for longer time periods but are changed quite often in order to monitor for complications [1]. A patient found to have been wearing the same cast for 28 months is extremely rare and there have been no previous cases reported in the literature. Patients who are known to be wear casts occasionally fail to attend for cast removal. In this scenario an awareness of the extent of potential complications is useful for this less compliant patient group.

Halanski and Noonan [2] reviewing plaster cast complications describe joint stiffness, muscle atrophy, cartilage degradation, ligament weakening and disuse osteoporosis. Joint stiffness was present in this case but was relatively insubstantial with only 10° of relative reduction in passive plantar flexion. This finding suggests that any stiffness observed after cast removal may be attributable to capsular stretch pain.

Muscle atrophy as a consequence of cast immobilization has been described [3] and was observed in this case where the leg circumference was substantially reduced. Research has attributed this change to an increase in both the resting inorganic phosphate concentration in skeletal muscle [4] and a change in the neural command of muscle contraction [5] with immobilization.

Skin complications have been described following plaster cast immobilization. Ulceration occurs where there is insufficient padding over bony protuberances and excoriation is known to occur particularly in casts worn by children which have become soiled [6]. One case describes skin atrophy and hyperpigmentation thought to be a variant of stasis dermatitis [7]. In this case the skin under the dense scales was relatively healthy. The small and regularly distributed pitted ulcers occurred where each individual skin pore had become blocked. The tissue at the base of these pits was healthy.

## Conclusion

Prolonged cast immobilization is extremely rare and occurs in non compliant patients. This case demonstrates muscle atrophy which was anticipated. The stiffness of the ankle joint was not marked. Skin changes were minor with no substantial areas of ulceration or stasis dermatitis. Where patients choose to remain in their cast for prolonged duration the complications may only be minor.

## Competing interests

The authors declare that they have no competing interests.

## Consent

Written informed consent was obtained from the patient for publication of this case report and any accompanying images. A copy of the written consent is available for review by the Editor-in-Chief of this journal.

## Authors' contributions

CFY and DWE reviewed the patient and performed the operation. SE researched for previous case reports and evidence. HI documented and described the findings. HI, SE and DWE contributed to the writing of the manuscript. All authors reviewed and approved the final manuscript.
